# Distinct clinical and immunologic patterns of interferon-γ release assays: implications for indeterminate results and sequential testing strategies

**DOI:** 10.1128/spectrum.02159-26

**Published:** 2026-09-21

**Authors:** Hyunji Kim, Jeong Eon Park, Sungmin Kim, Joon Hee Lee, Dong Woo Shin, Kyunghoon Lee, Yun Ji Hong, Kyoung Un Park

**Affiliations:** 1 Department of Laboratory Medicine, Seoul National University Bundang Hospital and Seoul National University College of Medicine 65462, Seoul, Republic of Korea; Central Texas Veterans Health Care System, Temple, Texas, USA

**Keywords:** interferon-γ release assays, sequential strategy, host-dependent immune phenotypes, T-SPOT, QFT-GP

## Abstract

Interferon-γ release assays (IGRAs) are widely used for tuberculosis (TB) infection testing and are often considered interchangeable. We evaluated whether differences between T-SPOT.TB (T-SPOT) and QuantiFERON-TB Gold Plus (QFT-GP) have clinically relevant implications, particularly for indeterminate results. In this prospective study, 703 patients with suspected TB infection underwent concurrent T-SPOT and QFT-GP testing. Performance was assessed across three reference strata—microbiologically confirmed disease, initiation of TB preventive treatment for presumed TB infection, and culture-confirmed nontuberculous mycobacterial disease as a specificity reference—and indeterminate results were analyzed using interferon-γ responses and host immune factors. T-SPOT showed higher positivity than QFT-GP in treated patients (77.8% vs 50.0%). Among 75 patients with indeterminate T-SPOT results (10.7%), QFT-GP provided determinate results in 89.3%, enabling clinical reclassification. Antigen-specific interferon-γ responses were significantly higher in QFT-GP–positive cases despite T-SPOT indeterminacy (*P* = 5.0 × 10⁻⁶). Mechanisms of indeterminacy differed: T-SPOT indeterminacy arose predominantly from near-threshold antigen responses (60.0%) and, in a minority, from failure of the assay control system, whereas QFT-GP indeterminacy was uniformly attributable to mitogen failure. T-SPOT resolved 61.9% of QFT-GP–indeterminate cases. IGRAs are not functionally interchangeable but, instead, provide complementary clinical information. Indeterminate results are mechanistically heterogeneous: near-threshold antigen responses were frequently resolved, and occasionally resolved as positive, by the alternative platform, whereas failure of the assay control system—particularly concurrent mitogen failure on both assays—marked host immune impairment. A sequential testing strategy—T-SPOT followed by QFT-GP in indeterminate cases—may improve diagnostic yield and reduce uncertainty in clinical practice.

**IMPORTANCE:**

Tuberculosis remains one of the leading infectious causes of death worldwide. Detecting people who carry *Mycobacterium tuberculosis* without symptoms is essential for preventing future disease, but currently available blood tests do not always provide clear answers. In this study of more than 700 patients, we found that two commonly used tuberculosis blood tests capture different aspects of the immune response and, therefore, do not always produce the same result. One test was more likely to identify infection when tuberculosis was clinically suspected, whereas the other often provided interpretable results when the first test was inconclusive. We also found that unclear results were strongly influenced by the patient’s immune status. These findings suggest that the two tests can be used together rather than viewed as interchangeable and that considering immune health may improve the interpretation of tuberculosis screening results and support more effective prevention strategies.

## INTRODUCTION

Tuberculosis (TB) remains a leading cause of death from a single infectious agent worldwide, and effective identification and treatment of TB infection (TBI) are central to TB control strategies (1). In clinical practice, diagnostic testing for TBI serves as a triage tool to guide TB preventive treatment (TPT) rather than as a definitive indicator of active disease (2).

Interferon-γ release assays (IGRAs), including T-SPOT.TB (T-SPOT) and QuantiFERON-TB Gold Plus (QFT-GP), are widely used for TB infection testing and have largely replaced the tuberculin skin test in Bacille Calmette-Guérin (BCG)-vaccinated populations because of their improved specificity (3). Despite differences in assay design, these platforms are generally regarded as interchangeable in current clinical guidelines.

However, indeterminate IGRA results remain a persistent and clinically unresolved problem, particularly in older adults and immunocompromised patients, in whom test interpretation directly influences treatment decisions (4–6). Indeterminate results are commonly attributed to technical failure, but they may also reflect underlying immune dysfunction, including impaired mitogen responsiveness, increased background reactivity, or reduced cellular immune reserve (7, 8). Current guidelines provide limited direction on how to interpret or manage indeterminate or discordant IGRA results, creating uncertainty in clinical decision-making (9).

The two platforms differ fundamentally in what they measure. T-SPOT is an enzyme-linked immunospot assay: peripheral blood mononuclear cells are isolated, standardized to a fixed input of 250,000 cells per well, and stimulated with ESAT-6 or CFP-10, and the read-out is the number of individual T cells secreting interferon-γ, enumerated as discrete spots. QFT-GP is an enzyme-linked immunosorbent assay: whole blood is drawn directly into antigen-coated tubes (TB1, eliciting predominantly CD4^+^ responses; TB2, additionally eliciting CD8^+^ responses) and incubated, after which the read-out is the total concentration of interferon-γ released into the harvested plasma, expressed in IU/mL. T-SPOT, therefore, enumerates responding cells at a standardized cell input, whereas QFT-GP quantifies secreted cytokine at a standardized blood volume; the two are not equivalent read-outs of the same biological quantity (10, 11). These differences suggest that the assays may capture distinct aspects of host immune function rather than measuring identical biological signals.

This raises an important clinical question: are these assays truly interchangeable, or do they provide complementary information that can be leveraged to improve diagnostic interpretation—particularly in indeterminate cases?

To address this, we conducted a prospective, head-to-head study in patients with suspected TB infection undergoing concurrent T-SPOT and QFT-GP testing. Concurrent testing with both platforms in all participants was performed for head-to-head comparison under the study protocol and does not reflect, and is not intended to recommend, routine clinical practice. We hypothesized that T-SPOT would show higher positivity in clinically supported infection (5, 12), QFT-GP would provide diagnostic clarification in T-SPOT–indeterminate cases (13, 14), and patterns of indeterminate results would reflect host-dependent immune status rather than random assay variability (4, 6, 15).

By examining assay complementarity and host-associated determinants of indeterminacy, we aimed to inform a clinically applicable sequential testing strategy for TB infection diagnosis.

## MATERIALS AND METHODS

We conducted a prospective comparative analysis of IGRAs between June and November 2025 in 703 patients with suspected TB infection who underwent simultaneous testing with T-SPOT and QFT-GP at a single-center. This study was approved by the Institutional Review Board of Seoul National University Bundang Hospital (no. B-2603-1031-101). Participants aged 5 years or older were eligible for inclusion. Blood samples from pediatric participants were obtained only when clinically indicated for routine diagnostic testing, including TB IGRA assays, and no additional blood collection was performed solely for research purposes. Given the minimal-risk nature of the study, verbal informed consent was obtained from the parents or legal guardians of minor participants in accordance with the approved study protocol and institutional ethical guidelines.

The study was structured around three predefined hypotheses:

- Assay Performance Hypothesis: IGRAs differ in their ability to detect clinically supported TB infection.
- Complementarity Hypothesis: Functional differences in assay control systems enable one IGRA to provide diagnostic clarification in cases deemed indeterminate by another.
- Host-Dependent Performance Hypothesis: Indeterminate outcomes reflect distinct patterns of immune functional failure influenced by host-related factors, including immunosuppression, age, and hematologic reserve.

To evaluate the Assay Performance Hypothesis, assay positivity rates were across three predefined reference strata: participants with culture- or PCR-confirmed *Mycobacterium tuberculosis*; participants who initiated TPT for presumed TB infection; and participants with culture-confirmed nontuberculous mycobacterial disease, included as a specificity reference. The assay demonstrating superior detection in these clinically supported populations was subsequently designated as the provisional anchor assay for downstream comparative analyses.

To test the Complementarity and Host-Dependent Performance Hypotheses, indeterminate results were categorized according to predefined functional failure patterns. T-SPOT was performed according to the institutional standard operating procedure. Peripheral blood mononuclear cells were isolated, standardized to 250,000 cells per well, and stimulated with Panel A (ESAT-6) or Panel B (CFP-10), alongside an AIM-V negative control and a phytohemagglutinin positive control. A result was reported as positive when Panel A or Panel B minus AIM-V exceeded 7 spots and as negative when both were below 5 spots, in each case with AIM-V below 10 spots and the positive control above 20 spots. A result was reported as indeterminate under any of three conditions: (i) high background, AIM-V ≥10 spots; (ii) inadequate positive control, ≤20 spots; or (iii) a near-threshold antigen response, Panel A or Panel B minus AIM-V equal to 5, 6, or 7 spots. Conditions (i) and (ii) reflect failure of the assay control system—respectively, non-specific reactivity that precludes reliable background subtraction, and failure of global T-cell activation that precludes a valid negative call. Condition (iii) reflects a quantitatively ambiguous antigen-specific response obtained under a valid control system and is, therefore, mechanistically distinct from control failure. Indeterminate results were analyzed both in aggregate, as reported to the requesting clinician, and stratified by these mechanisms.

QFT-GP results were interpreted according to the manufacturer’s algorithm. A result was positive when TB1 or TB2 minus nil was ≥0.35 IU/mL and ≥25% of the nil value, irrespective of the mitogen response; negative when antigen responses fell below this threshold and mitogen minus nil was ≥0.5 IU/mL; and indeterminate under either of two conditions—a nil value >8.0 IU/mL, indicating excessive background reactivity, or antigen responses below the positive threshold combined with mitogen minus nil <0.5 IU/mL, indicating failure of global T-cell activation. Both routes to indeterminacy were evaluated separately.

We further evaluated antigen-specific interferon-γ responses:

- Nil
- Antigen minus Nil
- Mitogen minus Nil

Immune status was defined by exposure to chemotherapy or immunosuppressive agents within 4 weeks before testing. Biological reserve was assessed by age group and pre-processing white blood cell counts measured during peripheral blood mononuclear cell preparation for T-SPOT.

All analyses were performed using KNIME Analytics Platform (version 5.9). Continuous variables were compared using the Mann–Whitney *U* test. Paired indeterminate outcomes were analyzed using McNemar’s exact test. Associations were evaluated using the *χ*² test or Fisher’s exact test. Logistic regression models were used to assess predictors of successful QFT-GP determination (rescue) among T-SPOT–indeterminate cases, defined as obtaining a determinate (positive or negative) QFT-GP result.

## RESULTS

### Provisional anchor assay for comparative analysis

A total of 703 patients with suspected TB infection underwent concurrent testing with T-SPOT and QFT-GP (Table 1). To anchor assay performance in clinically supported disease, we first examined patients who received TPT. Among the 36 treated individuals, T-SPOT yielded positive results in 77.8%, compared with 50.0% for QFT-GP (absolute difference, 27.8%). A similar pattern was observed in the subgroup of 22 patients with radiologic abnormalities on chest imaging, in whom positivity rates were 77.3% for T-SPOT and 54.5% for QFT-GP (absolute difference, 22.8%).

**TABLE 1:** Baseline characteristics of the 703 participants

Characteristic	Value
Age, years—median (IQR)	52 (34–65)
Range	9–94
Age group—*n* (%)	
<18 years	37 (5.3)
18–29 years	95 (13.5)
30–49 years	198 (28.2)
50–69 years	278 (39.5)
≥70 years	95 (13.5)
Sex—*n* (%)	
Male	410 (58.3)
Female	293 (41.7)
Underlying condition—*n* (%)	
Immune-mediated inflammatory disease	297 (42.2)
Malignancy	100 (14.2)
Chronic kidney disease, dialysis, or solid-organ transplantation	47 (6.7)
Suspected or previous TB or NTM disease	80 (11.4)
Healthy control or research volunteer	53 (7.5)
Other	126 (17.9)
Immunosuppressive exposure—*n* (%)	
Chemotherapy	59 (8.4)
Immunosuppressive therapy	190 (27.0)
Either	226 (32.1)
Coexisting infection—*n* (%)	
HIV seropositive	2 (0.3)
HBsAg positive	7 (1.0)
Hematologic indices—median (IQR)	
White blood cell count/µL	6,610 (5,215–8,375)
Absolute lymphocyte count/µL	1,805 (1,323–2,293)
PBMC preparation cell count/µL	13,080 (9,075–17,390)
Microbiological findings—*n* (**%**)	
Culture- or PCR-confirmed *M. tuberculosis*	10 (1.4)
Culture-confirmed nontuberculous mycobacteria	20 (2.8)
TB-directed drug therapy—*n* (**%**)	
Any	36 (5.1)
Treatment for active TB disease	17 (2.4)
TB preventive treatment	14 (2.0)
NTM-directed therapy	2 (0.3)
Regimen not established	3 (0.4)
IGRA result—*n* (%)	
T-SPOT positive/negative/indeterminate	137 (19.5)/491 (69.8)/75 (10.7)
QFT-GP positive/negative/indeterminate	104 (14.8)/578 (82.2)/21 (3.0)

In addition, assay positivity was examined across three reference strata. Among participants with culture- or PCR-confirmed *M. tuberculosis* (*n* = 10), T-SPOT was positive in 9 (90.0%, 95% CI 55.5–99.7) and QFT-GP in 5 (50.0%, 18.7–81.3). Among participants receiving TPT for presumed infection (*n* = 14), T-SPOT was positive in 11 (78.6%, 49.2–95.3) and QFT-GP in 7 (50.0%, 23.0–77.0). Among participants with culture-confirmed nontuberculous mycobacterial disease, included as a specificity reference (*n* = 20), T-SPOT was positive in 5 (25.0%, 8.7–49.1) and QFT-GP in 4 (20.0%, 5.7–43.7). Across the treated subgroup as a whole, 10 participants were T-SPOT–positive but QFT-GP–negative, and none showed the reverse pattern (exact McNemar *P* = 0.002). Higher T-SPOT positivity was, therefore, observed consistently across both TB reference strata, while positivity in the nontuberculous mycobacterial stratum was low for both platforms, consistent with the antigen specificity of ESAT-6 and CFP-10. Given the size of each stratum, these estimates are imprecise and are presented as exploratory.

### Complementarity hypothesis: QFT-GP provides diagnostic rescue in T-SPOT–indeterminate cases

**Fig 1:**
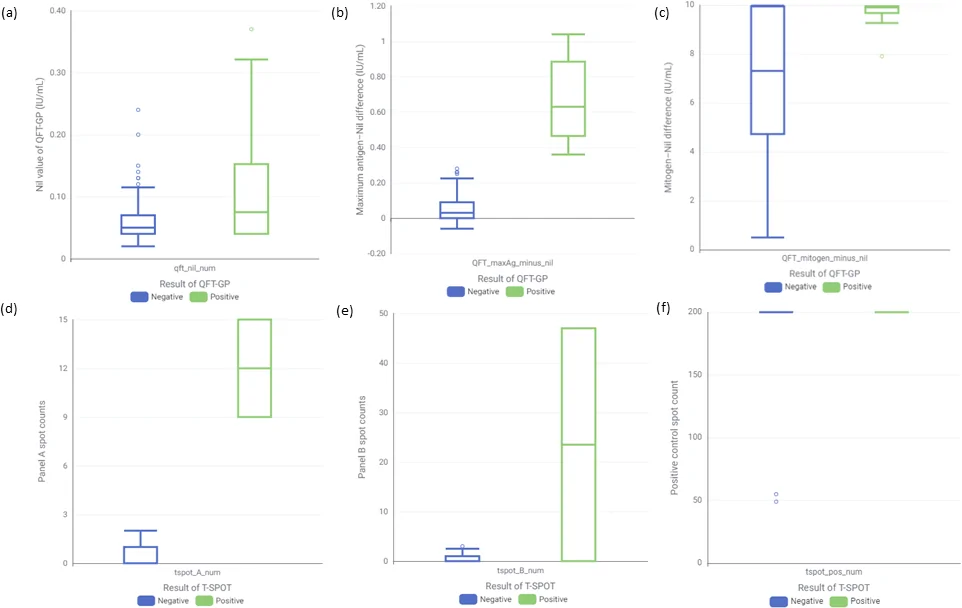
Comparison of QFT-GP and T-SPOT tube responses in reciprocal rescue analyses. (**a–c**) Among samples with indeterminate T-SPOT.TB (T-SPOT) results, the performance of individual QuantiFERON-TB Gold Plus (QFT-GP) tubes was evaluated by comparing (**a**) Nil concentrations, (**b**) maximum antigen–Nil differences, and (**c**) mitogen–Nil differences between samples yielding positive and negative QFT-GP results. The control-related measures—(**a**) Nil and (**c**) mitogen–Nil difference—showed no significant differences between groups, whereas the result-reflecting measure—(**b**) maximum antigen–Nil difference—demonstrated a statistically significant difference between QFT-GP–positive and QFT-GP–negative results. (**d–f**) In the reciprocal rescue analysis, samples with indeterminate QFT-GP results were evaluated using T-SPOT tube responses. Spot counts were compared between T-SPOT–positive and T-SPOT–negative results for (**d**) Panel **a** (ESAT-6–stimulated well—AIM-V control), (**e**) Panel **b** (CFP-10–stimulated well— AIM-V control), and (**f**) positive control spot counts.

Within the overall cohort, 75 patients (10.7%) had indeterminate T-SPOT results (Table 2). Among the 75 T-SPOT indeterminate results, 45 (60.0%) were attributable to a near-threshold antigen response, 14 (18.7%) to inadequate positive control, 12 (16.0%) to high background, and 1 (1.3%) to both control failures; in 3 (4.0%), the panel spot counts were not retrievable (Table 3). Control failure, therefore, accounted for 27 cases, or 3.8% of the cohort. QFT-GP yielded a determinate result in 43 of 45 near-threshold cases (95.6%), 11 of 12 high-background cases (91.7%), 11 of 14 low-positive-control cases (78.6%), and neither the single dual-failure case (0%), a gradient tracking the integrity of the T-SPOT control system. All eight cases reclassified as QFT-GP–positive arose from the near-threshold group, whereas cases attributable to control failure were uniformly reclassified as negative. Consistent with this, no participant had a nil value exceeding 8.0 IU/mL (highest observed 2.8 IU/mL), and all QFT-GP indeterminate results were attributable to mitogen failure. To determine whether QFT-GP determinations reflected meaningful immune discrimination rather than technical artifact, antigen-specific interferon-γ responses were examined. Among T-SPOT–indeterminate cases, antigen-specific interferon-γ responses (antigen minus Nil) were significantly higher in QFT-GP–positive than in QFT-GP–negative samples (*P* = 5.047 × 10⁻⁶), while mitogen responses remained functionally adequate in both groups (Fig. 1a through c). This pattern supports the biological plausibility of QFT-GP-based reclassification and suggests preservation of antigen-specific responsiveness despite T-SPOT indeterminacy.

**TABLE 2:** Comparison of T-SPOT and QFT-GP results in 703 patients^*a*^^,^^*b*^

		QFT-GP			Total
		Positive	Negative	Indeterminate	
T-SPOT	Positive	91 (12.9)	44 (6.3)	2 (0.3)	137 (19.5)
	Negative	5 (0.7)	475 (67.6)	11 (1.6)	491 (69.8)
	Indeterminate	8 (1.1)	59 (8.4)	8 (1.1)	75 (10.7)
	Total	104 (14.8)	578 (82.2)	21 (3.0)	703 (100.0)

^*a*^ Values are *n* (%) of the total cohort (*n* = 703). Overall agreement 81.7%; agreement among pairs determinate by both assays (*n* = 615) 92.0%; Cohen’s *κ* = 0.74. Discordance was asymmetric (44 vs 5; McNemar, *P* < 0.001).

^*b*^ T-SPOT, T-SPOT.TB; QFT-GP, QuantiFERON-TB Gold Plus.

**TABLE 3:** Mechanism of T-SPOT.*TB* indeterminacy and corresponding QuantiFERON-TB Gold Plus results (*n* = 75)^*a*^^,^^*b*^

Mechanism of T-SPOT indeterminacy	No. (%) of results	QFT-GP positive	QFT-GP negative	QFT-GP indeterminate	Determinate by QFT-GP, no. (% [95% CI])
Near-threshold antigen response (panel A or B minus nil = 5–7 spots)	45 (60.0)	8	35	2	43 (95.6 [84.9–99.5])
Control system failure	27 (36.0)	0	22	5	22 (81.5 [61.9–93.7])
Inadequate positive control (≤20 spots)	14 (18.7)	0	11	3	11 (78.6 [49.2–95.3])
High background (nil ≥ 10 spots)	12 (16.0)	0	11	1	11 (91.7 [61.5–99.8])
Both of the above	1 (1.3)	0	0	1	0 (0 [0–97.5])
Panel spot counts unavailable	3 (4.0)	0	2	1	2 (66.7 [9.4–99.2])
Total	75 (100)	8 (10.7)	59 (78.7)	8 (10.7)	67 (89.3 [80.1–95.3])

^*a*^ T-SPOT, T-SPOT.TB; QFT-GP, QuantiFERON-TB Gold Plus.

^*b*^ Results other than those attributed to control system failure had background and positive control responses within specification and correspond to the results recorded as “Normal” in Table 4 (*n* = 48).

**Fig 2:**
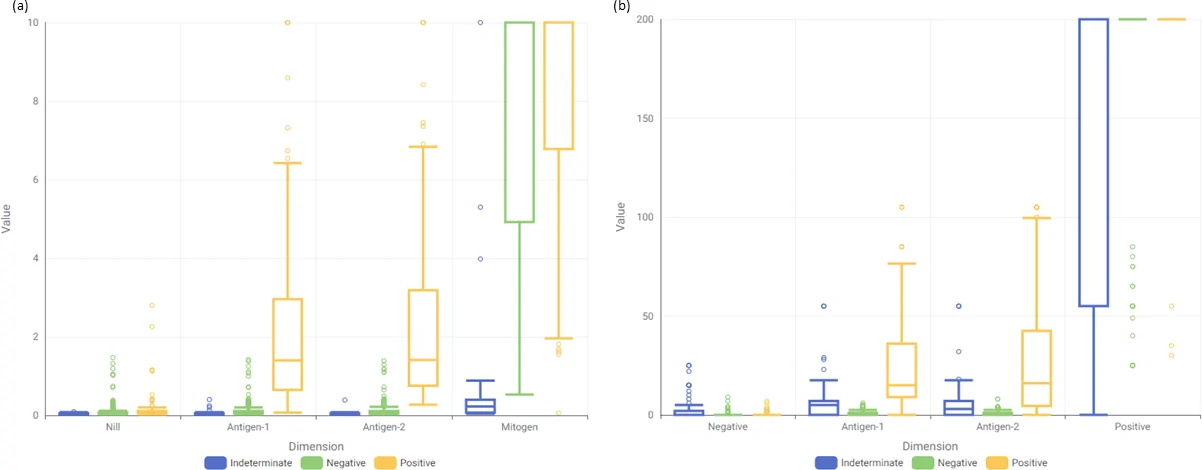
Distinct control-response mechanisms underlying indeterminate results across all participants. Box-and-whisker plots show tube-specific response distributions stratified by final test interpretation (indeterminate, negative, and positive) in the overall study population. (**a**) In QuantiFERON-TB Gold Plus, indeterminate results are predominantly characterized by insufficient mitogen responses, indicating failure of the positive control, while Nil tube values remain low, arguing against a high background–driven mechanism. Antigen-specific responses (Antigen-1 and Antigen-2) are minimal in these cases. (**b**) In T-SPOT.TB, indeterminate results reflect heterogeneous patterns of functional immune failure, including reduced positive control responses and, in a subset, increased background activity, in contrast to antigen-driven responses observed in positive results. Boxes represent the interquartile range with median values, whiskers indicate the range, and dots indicate outliers.

Distinct mechanisms of assay failure emerged across platforms (Fig. 2). Mechanisms of indeterminacy differed markedly between platforms. T-SPOT indeterminacy was heterogeneous, arising predominantly from near-threshold antigen responses (60.0%) and, in a minority, from failure of the assay control system (36.0%). By contrast, QFT-GP indeterminacy was uniformly attributable to mitogen failure. Among the small subset of cases indeterminate by both assays (*n* = 8), mitogen failure was universal, and 62.5% of these patients had end-stage renal disease or related comorbidities, consistent with profound functional immune impairment (Table 4). Reciprocal rescue analysis further supported nonredundant assay behavior. Among the 21 samples with indeterminate QFT-GP results, T-SPOT yielded determinate results in 13 cases (61.9%), including both positive and negative classifications (Fig. 1d and e). The remaining 8 cases (38.1%) remained indeterminate by both assays. Although the magnitude of rescue was lower than that observed for QFT-GP in T-SPOT–indeterminate cases, these findings indicate that T-SPOT can also resolve a substantial subset of inconclusive QFT-GP outcomes.

**TABLE 4:** Representative cases of indeterminate results on two IGRAs: T-SPOT and QFT-GP^*b*^

No	Sex	Age (*y*)	WBC count (/μL)	Neutrophils (%)	Lymphocytes (%)	Monocytes (%)	ALC (/μL)	Diagnosis	TPT	CTx	IST	Cause of T-SPOT–indeterminate result	Cause of QFT-GP–indeterminate result
**1**	F	9	11,680	89.9	8.7	0.9	1,016	Ulcerative colitis	N	N	Y	Other cause	Low mitogen
**2**	M	41	9,080	95.1	1.7	3.2	154.4	IgA nephropathy	N	N	N	High background	Low mitogen
**3**	M	64	15,940	NA	NA	NA	NA	Kidney transplant status^*a*^	N	N	Y	Low positive control	Low mitogen
**4**	F	65	7,130	97.8	1	1.1	71.3	ADPKD and ESRD	N	N	N	Low positive control	Low mitogen
**5**	F	66	26,660	79.2	8.2	10.2	2,186	Multiple myeloma	N	Y	Y	High background	Low mitogen
**6**	M	67	8,260	78.8	18	1.9	1,487	DM with ESRD	N	N	N	Other cause	Low mitogen
**7**	F	70	12,410	97.9	1.1	0.9	136.5	DM with ESRD	N	N	N	Low positive control	Low mitogen
**8**	M	73	13,730	84.6	5.8	9.5	796.3	Herpes simplex encephalitis	N	N	Y	Other cause	Low mitogen

^*a*^ The patient underwent kidney transplantation 3 weeks before the IGRA test.

^*b*^ IGRA, interferon-γ release assay; T-SPOT, T-SPOT.TB; QFT-GP, QuantiFERON-TB Gold Plus; F, female; M, male; WBC, white blood cell; ALC, absolute lymphocyte count; TPT, TB preventive treatment; CTx, chemotherapy; IST, immunosuppressive therapy; ADPKD, autosomal dominant polycystic kidney disease; ESRD, end-stage renal disease; DM, diabetes mellitus; NA, not available; N, no; Y, yes.

Taken together, these reciprocal analyses demonstrate asymmetric complementarity between the two IGRA platforms: QFT-GP more frequently rescued T-SPOT–indeterminate results, whereas T-SPOT resolved a meaningful proportion of QFT-GP–indeterminate cases.

### Assay indeterminacy reflects host-dependent immune status

Host immune status further modulated assay performance (Table 5). Among immunocompromised patients (*n* = 226), T-SPOT produced more indeterminate results than QFT-GP (30 vs 13 indeterminate results; 13.3% vs 5.8% overall), a difference that was statistically significant by McNemar’s exact test (*P* = 0.005). This asymmetry suggests that QFT-GP may retain relatively greater functional resilience under moderate immune suppression when both assays are performed concurrently. However, within the T-SPOT–indeterminate subgroup, the QFT-GP outcome distribution did not differ by immunosuppression status, indicating that once T-SPOT fails, rescue capacity by QFT-GP is not strongly modified by this binary immune classification. Age was significantly associated with T-SPOT–indeterminate rates (Table S1). Indeterminate rates increased progressively across age strata, reaching 21.9% among individuals aged ≥70 years (*P* < 0.001). By contrast, age was not independently associated with QFT-GP rescue success in multivariable models. Instead, baseline hematologic reserve appeared more informative (Table S2). Among patients with T-SPOT–indeterminate results, those with low white blood cell counts had higher odds of obtaining determinate results with QFT-GP (adjusted OR ≈ 4.86) although this association did not reach conventional statistical significance (*P* = 0.066). Age-adjusted analyses suggested that leukocyte status, rather than chronological age, showed a trend toward association but did not reach statistical significance.

**TABLE 5:** Immune status–dependent functional mechanisms associated with determinate and indeterminate T-SPOT and QFT-GP results^*a*^^,^^*b*^

T-SPOT	Immune status	QFT-GP result	Mitogen responses in QFT-GP	Control and background responses in T-SPOT	No.
Determinate	Immunosuppressed	Determinate	High	Normal	187
		Indeterminate	Low	Normal	9
	Non-immunosuppressed	Determinate	High	Normal	427
			Low	Normal	1
		Indeterminate	Low	Normal	4
Indeterminate	Immunosuppressed	Determinate	High	High background	8
				Inadequate positive control	6
				Normal (near-threshold antigen response)	12
		Indeterminate	Low	High background	1
				Inadequate positive control	1
				Normal (near-threshold antigen response)	2
	Non-immunosuppressed	Determinate	High	High background	3
				Inadequate positive control	5
				Normal (near-threshold antigen response)	33
		Indeterminate	Low	High background and inadequate positive control	1
				Inadequate positive control	2
				Normal (near-threshold antigen response)	1
Total					703

^*a*^ Immunosuppression was defined as receipt of chemotherapy or immunosuppressive therapy. Mitogen response is classified as low when the mitogen-minus-nil value was <0.5 IU/mL, the manufacturer criterion for an indeterminate result; all 21 QuantiFERON-TB Gold Plus indeterminate results met this criterion, and no participant had a nil value >8.0 IU/mL. “Normal” denotes background and positive control responses within specification; in these results, indeterminacy arose from a near-threshold antigen response (panel A or B minus nil = 5–7 spots), except in three results in which the panel spot counts could not be retrieved (one immunosuppressed with an indeterminate QFT-GP result and two non-immunosuppressed with determinate QFT-GP results). Mechanisms of T-SPOT indeterminacy are shown in full in Table 2.

^*b*^ T-SPOT, T-SPOT.TB; QFT-GP, QuantiFERON-TB Gold Plus.

Notably, in cases where T-SPOT indeterminacy was attributable to high background reactivity, QFT-GP provided determinate results in 91.7% of instances. This finding suggests that background-driven T-SPOT instability does not necessarily imply global T-cell failure and that whole-blood stimulation assays may mitigate this specific vulnerability. Collectively, these results suggest that T-SPOT and QFT-GP are not fully interchangeable diagnostic tools but, instead, interrogate partially distinct dimensions of host cellular immunity. T-SPOT demonstrated higher positivity in clinically supported infection, whereas QFT-GP showed greater robustness in certain contexts of immune perturbation and background instability. Patterns of indeterminate results were mechanistically distinct and host-dependent, suggesting that assay performance may reflect underlying immune architecture rather than solely technical variability.

## DISCUSSION

In this prospective head-to-head study, we show that T-SPOT and QFT-GP are not clinically interchangeable assays but, instead, provide complementary diagnostic information, particularly in patients with indeterminate results. Rather than functioning as redundant diagnostic tools, our data indicate that the two assays capture partially overlapping yet biologically distinct dimensions of host cellular immune responses, resulting in platform-specific patterns of positivity and indeterminacy.

In patients receiving treatment for clinically supported TBI, T-SPOT demonstrated higher positivity than QFT-GP. Although the microbiologically confirmed subgroup was small, which limits the precision of sensitivity estimates (9, 16), this finding supports the use of T-SPOT as a clinically sensitive initial test in selected populations. Importantly, this should not be interpreted as universal superiority but, rather, as evidence of context-dependent performance differences between platforms (17).

A key finding of this study is that QFT-GP provided determinate results in the majority of T-SPOT–indeterminate cases, enabling clinically actionable reclassification in nearly 90% of patients. Antigen-specific interferon-γ responses remained significantly discriminative despite T-SPOT indeterminacy, while mitogen responses were preserved, supporting the biological validity of these reclassifications. These results suggest that indeterminate T-SPOT outcomes, particularly those arising from near-threshold antigen responses or high background, do not necessarily indicate global T-cell dysfunction and can be resolved by an alternative platform (12–14).

Reciprocal analysis provided additional insight into the relationship between platforms. Among QFT-GP–indeterminate samples, T-SPOT produced determinate results in 61.9% of cases. Taken together, these bidirectional rescue patterns reinforce the concept that discordant or indeterminate results are not random but, instead, reflect systematic differences in assay architecture and immune readouts. Importantly, this reciprocal rescue analysis addressed a different question from that of the anchor assay comparison. While anchor designation was based on relative positivity in clinically supported infection, rescue analyses examined whether one platform could provide an interpretable classification when the other failed.

### Indeterminate results as functional immune phenotypes

A key conceptual implication of this study is that indeterminate IGRA results may encode biologically relevant information rather than representing purely technical failure. T-SPOT indeterminacy was heterogeneous with near-threshold antigen responses or failure of the assay control system, whereas QFT-GP indeterminacy was uniformly attributable to mitogen failure. These distinct patterns suggest platform-specific vulnerabilities under different immune conditions.

When indeterminacy occurred across both assays, mitogen failure was universal. In this small subgroup, several patients had advanced renal disease, transplant status, or other immune-modifying comorbidities. This convergence strongly supports the interpretation that dual-platform indeterminacy represents a clinically meaningful marker of profound T-cell hyporesponsiveness rather than coincidental assay failure (18).

End-stage renal disease and related conditions are known to be associated with uremia-related immune dysfunction, including impaired T-cell proliferation and reduced cytokine production (19). Prior studies in dialysis populations have reported increased indeterminate IGRA rates, frequently driven by mitogen failure (15, 20, 21). Our findings are consistent with this framework: while QFT-GP rescued many T-SPOT–indeterminate cases overall, dual-platform indeterminacy tended to occur in patients with advanced renal dysfunction or substantial immune perturbation. These findings highlight that IGRA results—particularly indeterminate outcomes—should be interpreted within the broader clinical and immunologic context rather than in isolation.

### Host immune status and platform-specific vulnerability

Host characteristics further modulated assay performance. T-SPOT–indeterminate rates increased significantly with age in our study, consistent with immunosenescence and altered immune activation thresholds (4, 6). However, chronological age alone did not independently predict QFT-GP rescue success. Instead, baseline leukocyte status showed a trend toward association with QFT-GP determinate outcomes following T-SPOT indeterminacy although this did not reach statistical significance.

This observation suggests that functional immune reserve, rather than chronological aging *per se*, may be a more relevant determinant of IGRA performance. ELISPOT-based assays may be more sensitive to perturbations in background reactivity or control responses in aging immune systems (22, 23), whereas whole-blood cytokine quantification may retain relative stability under certain forms of moderate immunosuppression (24, 25). Notably, among immunocompromised patients, QFT-GP produced fewer indeterminate results than T-SPOT. However, once T-SPOT yielded an indeterminate result, QFT-GP rescue capacity was not strongly modified by immunosuppression status, suggesting that mechanisms of indeterminacy are multifactorial and not reducible to a single clinical label.

### Implications for IGRA deployment in BCG-vaccinated populations

In regions with high BCG vaccination coverage, IGRAs are preferred over tuberculin skin testing because of superior antigen specificity (26, 27). Current guidelines generally treat available IGRA platforms as broadly interchangeable (9). However, our findings suggest that this assumption may oversimplify clinically relevant differences between assays, particularly in populations with heterogeneous immune status.

Structural differences between T-SPOT (cell-based enumeration) and QFT-GP (whole-blood cytokine quantification) appear to confer complementary strengths (11, 28). In this cohort, T-SPOT showed higher positivity in clinically supported infection, whereas QFT-GP demonstrated relative robustness in the setting of background instability and selected forms of immune perturbation (22, 23).

**Fig 3:**
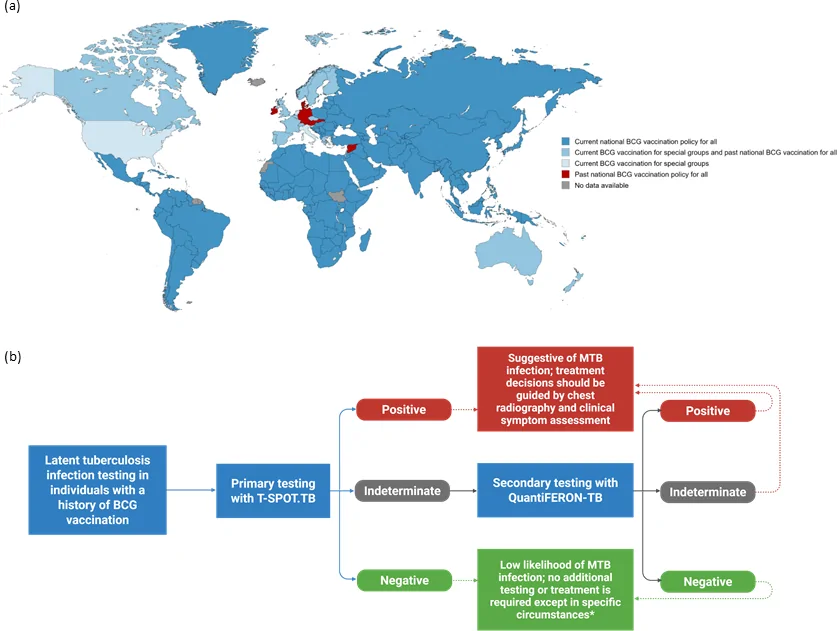
Proposed diagnostic workflow for the identification of tuberculosis infection. (**a**) Global map of BCG vaccination practices worldwide. The map was created by the authors using PaintMyMap (paintmymap.com) based on BCG vaccination policy data obtained from the BCG World Atlas (bcgatlas.org). (**b**) In accordance with WHO guidelines, IGRAs are preferentially used in individuals with prior BCG vaccination. This pathway assumes laboratory capacity for both platforms. T-SPOT.TB is used as the primary test, and QuantiFERON-TB is performed as a secondary test in cases with indeterminate T-SPOT.TB results. *Immunocompromised individuals, recent exposure to tuberculosis (window period), or strong clinical suspicion. Abbreviations: WHO, World Health Organization; BCG, Bacille Calmette-Guérin; IGRA, interferon-γ release assay; MTB, *Mycobacterium tuberculosis.*

From a clinical perspective, these findings support a potential role for a sequential or complementary testing strategy rather than strict assay substitution. Primary testing with T-SPOT may enhance detection in clinically suspected infection, while QFT-GP may serve as a complementary tool when T-SPOT results are indeterminate, particularly where indeterminacy arises from near-threshold antigen responses or high background (Fig. 3). Two practical qualifications follow. First, concurrent testing with both platforms is not warranted as routine practice. For immunocompetent individuals, a single IGRA is sufficient, and a second assay is of value in two circumstances: as a reflex test when the first returns an indeterminate result, which occurred in 10.7% of T-SPOT and 3.0% of QFT-GP assays in this cohort, and in patients with impaired immune function, in whom the platforms diverged most—immunosuppression reduced QFT-GP positivity from 17.4% to 9.3% (*P* = 0.004) while leaving T-SPOT positivity essentially unchanged (19.9% to 18.6%, *P* = 0.76), and QFT-GP indeterminacy rose from 1.7% to 5.8% (*P* = 0.007). Second, where laboratory capacity allows only one platform, the choice should take account of the local burden of tuberculosis as well as of logistics. T-SPOT requires cell isolation, a density-gradient step, cell counting, and same-day processing, whereas QFT-GP requires only incubation of whole-blood tubes; but the difference in sensitivity between the platforms is a property of the assays, whereas the number of infections that difference uncovers depends on how many are present. The absolute yield of the more sensitive platform, therefore, rises with pre-test probability while its cost per test does not. In the present cohort, drawn from a population with an estimated incidence of 38 per 100,000 (29), T-SPOT classified 137 participants (19.5%) as positive compared with 104 (14.8%) by QFT-GP—33 additional positive results per 703 tested—and in a lower-burden population, the same difference in sensitivity would identify correspondingly fewer. We, therefore, report these estimates to inform that judgment locally rather than to prescribe a single algorithm. Such an approach aligns with a precision diagnostic framework, in which assay selection is tailored to the patient’s immune context rather than applied uniformly. Prospective validation in diverse epidemiologic settings will be required before broad implementation can be recommended.

A third qualification concerns feasibility. Sequential testing cannot be run on a single specimen: T-SPOT is read on the day after collection, by which time the interval for incubating QFT-GP tubes has elapsed, and T-SPOT itself requires viable cells processed on the day of draw. A second specimen is, therefore, needed from those whose first assay is indeterminate. Three considerations bound this burden. It applies to a minority—10.7% of participants under a T-SPOT-first algorithm, approximately one patient in nine. It is not an additional burden relative to current practice, in which an indeterminate result already prompts repeat testing; what changes is which assay is performed on the second specimen. And substitution is more productive than repetition, resolving 89.3% of indeterminate T-SPOT and 61.9% of indeterminate QFT-GP results, whereas repeating the same platform would not address inadequate positive control or mitogen failure, which reflect properties of the patient rather than of the specimen. Per 100 patients tested, the algorithm would generate approximately 11 repeat specimens and about 10 results that would otherwise have remained non-actionable. Against this, the strategy adds days to the diagnostic pathway, which matters where decisions are time-sensitive, and we did not measure turnaround time or cost; prospective evaluation of yield, turnaround, and cost-effectiveness is, therefore, required before adoption.

### Limitations

Several limitations merit consideration. First, the designation of an operational anchor assay was based on relatively small reference strata; although a microbiologically confirmed subgroup was available within the cohort, its size limits the precision of sensitivity comparisons. Second, immune status classification relied on clinical exposure to chemotherapy or immunosuppressive therapy and peripheral leukocyte indices, which may not fully capture functional T-cell competence. Third, this study was conducted in a single tertiary center in a country of intermediate tuberculosis burden; the absolute diagnostic yield reported here would be smaller in lower-burden settings. And assay performance may vary across settings with differing TB prevalence and comorbidity profiles. Finally, mechanistic inferences regarding immune architecture remain hypothesis-generating and require validation in studies incorporating detailed functional immunophenotyping.

### Conclusions

Rather than asking which IGRA is universally superior, our findings suggest a more clinically relevant question: Which assay most accurately reflects the host immune context at the time of testing? T-SPOT and QFT-GP appear to probe overlapping but nonidentical aspects of cellular immunity. Indeterminate results, far from being mere technical inconveniences, may provide insight into underlying immune reserve and activation state.

Importantly, these findings have practical implications for TB screening strategies, particularly in immunocompromised or diagnostically challenging populations, where assay selection may influence clinical decision-making. In an era increasingly oriented toward precision diagnostics, TB infection testing may benefit from host-adapted deployment strategies that acknowledge platform-specific biology. Future studies integrating immunologic profiling with clinical outcomes will be essential to translate these observations into guideline-level recommendations.

## Data Availability

The data supporting the findings of this study are available from the corresponding author upon reasonable request.

## SUPPLEMENTAL MATERIAL

Supplemental tables spectrum.02159-26-s0001.pdf Tables S1 and S2.

ASM does not own the copyrights to Supplemental Material that may be linked to, or accessed through, an article. The authors have granted ASM a non-exclusive, world-wide license to publish the Supplemental Material files. Please contact the corresponding author directly for reuse.
