# Supplementary material for: Distinct clinical and immunologic patterns of interferon-γ release assays: implications for indeterminate results and sequential testing strategies
Source: Microbiol Spectr. 2026 Sep 21;14(11):e02159-26. doi: 10.1128/spectrum.02159-26 (PMC13729163; doi:10.1128/spectrum.02159-26)
Supplement: Supplementary file 1 [file spectrum.02159-26-s0001.pdf]

Supplementary Materials

Supplementary Table 1. Determinate and indeterminate results of T-SPOT.TB (T-SPOT) and QuantiFERON-TB Gold Plus (QFT-GP) according to age group

Age group	T-SPOT	QFT-GP	No.
**≤29**	Determinate	Determinate	121
		Indeterminate	1
	Indeterminate	Determinate	9
**30-49**	Determinate	Determinate	187
		Indeterminate	2
	Indeterminate	Determinate	8
		Indeterminate	1
**50-69**	Determinate	Determinate	234
		Indeterminate	8
	Indeterminate	Determinate	32
		Indeterminate	4
**≥70**	Determinate	Determinate	73
		Indeterminate	2
	Indeterminate	Determinate	18
		Indeterminate	3
**Total**			703

Supplementary Table 2. Determination status of T-SPOT.TB (T-SPOT) and QuantiFERON-TB Gold Plus (QFT-GP) according to white blood cell (WBC) count

WBC counts	T-SPOT	QFT-GP	No.
Adequate	Determinate	Determinate	217
		Indeterminate	8
	Indeterminate	Determinate	28
		Indeterminate	6
Low	Determinate	Determinate	398
		Indeterminate	5
	Indeterminate	Determinate	39
		Indeterminate	2
**Total**			703
